# The Role of Earlier Receipt of a Lower Limb Prosthesis on Emergency Department Utilization

**DOI:** 10.1002/pmrj.12504

**Published:** 2020-12-11

**Authors:** Taavy A. Miller, Rajib Paul, Melinda Forthofer, Shane R. Wurdeman

**Affiliations:** ^1^ Department of Public Health Sciences University of North Carolina at Charlotte Charlotte NC; ^2^ Department of Clinical and Scientific Affairs Hanger Clinic Austin TX; ^3^ Department of Biomechanics University of Nebraska at Omaha Omaha NE

## Abstract

**Introduction:**

Adverse events after a lower limb amputation (LLA) can negatively affect the rehabilitation process and may lead to emergency department (ED) visits. Earlier receipt of a prosthesis, as compared to delayed or not receiving a prosthesis, may decrease or moderate the risk of increased ED utilization. In addition, adverse events (ie, fall‐related injury [FRI]) may be associated with increased health care utilization as measured by ED use. The implication of the timing of prosthesis provision after amputation and reduced ED use is not well established. Obtaining data about ED utilization early post‐LLA could assist the rehabilitation team in ensuring timely and appropriate access to improve outcomes.

**Objective:**

To determine the role that timing of prosthesis receipt has in ED utilization and the association of fall/FRI with health care utilization.

**Design:**

Retrospective observational cohort using commercial claims data. A logistic regression model was used to assess factors that influence ED utilization post‐LLA.

**Setting:**

Watson/Truven administrative database 2014 to 2016.

**Participants:**

The study sample consisted of 510 adults age 18 to 64 years with continuous enrollment for 3 years.

**Interventions:**

Independent variables included age, sex, diabetes status, amputation level, fall diagnosis, and prosthesis receipt. Fall was defined as presence of a diagnosis code in any outpatient procedure after the amputation date.

**Main Outcome Measure:**

ED use after amputation was defined as the presence of procedure codes that billed for ED services (99281 to 99285).

**Results:**

Individuals who receive a prosthesis early, within 0 to 3 months, post‐LLA were 48% (odds ratio [OR] 0.52, 95% confidence interval [CI] 0.28 to 0.97) less likely to use the ED compared to those who did not receive a prosthesis. Individuals who experienced a fall/FRI had 2.8 (OR 2.86, 95% CI 1.23 to 6.66) times the odds of ED utilization.

**Conclusion:**

Receipt of a prosthesis reduces the risk of ED use. The current study underscores the value of prostheses during the rehabilitation process after LLA.

## Introduction

The potentially preventable use of emergency departments (EDs) accounts for $38 billion dollars of spending waste within the health care industry in the United States.^1^ Annually about 20% of adults in the United States will visit the ED for acute care, which accounts for 141.4 million visits.^1^ ED utilization can be considered a proxy measure for increased health care utilization, which is associated with increased economic burden.^2^ Approximately 34% of total ED visits consist of individuals who have limitations in their activities of daily living (ADLs).^3^ In addition, those with functional limitations have nearly three times the odds of repeated visits among patients released from the ED.^4^ Understanding the population subgroups that are utilizing emergency services frequently, and in what manner, may inform targeted interventions to eliminate the avoidable expenses associated with nonemergent care.

Lower limb amputation (LLA) is a life‐changing event that requires an increased use of health care services, encompassing the amputation surgery, immediate postoperative care, rehabilitation, fitting of a prosthesis, and follow‐ups with other services after discharge into the community. Previous studies report that a shorter time between amputation surgery and receipt of a prosthesis improves use of and satisfaction with the device.^5^, ^6^ There is also evidence that increased ED use is associated with a poor quality of life (QoL).^7^ However, there is no certainty about the influence of early prosthesis receipt on ED use and potentially preventable health care utilization. Early mobility along with functional independence and ambulation is associated with reduced unnecessary health care utilization, whereas an increased risk of clinical complications is associated with no prosthesis.^8^ It has been suggested among community‐dwelling adults that falls or fall‐related injuries (FRIs) may be the result of functional limitation and poor physical activity.^2^, ^3^

Individuals with LLA are at a high risk of falls and FRI, with more than half of individuals with LLA reporting a fall at least once per year.^9^, ^10^ A fall or FRI after an amputation can negatively affect the rehabilitation process and may lead to increased health care utilization such as an outpatient medical visit, an ED visit, hospitalization, or admission to a long‐term care facility.^9^ A fall/FRI also results in pain, the need for medical treatment, increased fear of falling again, and self‐induced isolation or activity reduction, which leads to a reduced QoL.^11^ However, a percentage of emergency care or acute injuries incurred may be avoided with interventions such as access to a prosthesis. If prosthetic services result in cost avoidance by reducing preventable use of the ED, or improve function and prevent adverse events, then there is increased value placed on the associated health service such as receipt of a prosthesis.^8^, ^12^, ^13^

Earlier receipt of a prosthesis, as compared to delayed receipt or not receiving a prosthesis within 12 months after amputation surgery, may decrease the risk of future adverse events or increased health care utilization, and thereby contribute to improved patient outcomes and the value of having a prosthesis fit after amputation. The purpose of the present study was to determine the role that timing of prosthesis receipt has in ED utilization and the association of fall/FRI with health care utilization measured by ED use among adults who recently had a LLA in the United States. Two hypotheses were tested: (1) the receipt of a prosthesis and timing of intervention with a prosthesis reduces ED use and health care utilization, and (2) the receipt of a prosthesis and adverse events (ie, fall or FRI) may be factors associated with increased health care utilization among adults with LLA.

## Methods

### Study Design and Data Source

This was a retrospective observational cohort analysis using data extracted from the International Business Machines (IBM) Corporation Watson/Truven administrative (Watson) database. The database is populated annually from approximately 350 payers providing commercial (private insurance) claims (billing data) for their members. Data contain de‐identified longitudinal, patient‐level records. Included within the data set are all fully adjudicated claims for approximately 230 million unique individual patients in the United States. This included any orthotic/prosthetic services as well as all other inpatient and outpatient claims. The subset of individuals extracted was limited to either persons who received an amputation or orthotic/prosthetic services enrolled from 1 January 2014 through 31 December 2016. The original database as maintained by IBM Watson is de‐identified in nature and complies with the Health Insurance Portability and Accountability Act (HIPAA). The subsequent analysis is not considered human subject research and therefore does not require institutional review board (IRB) review or approval.

### Participants

**Figure 1:**
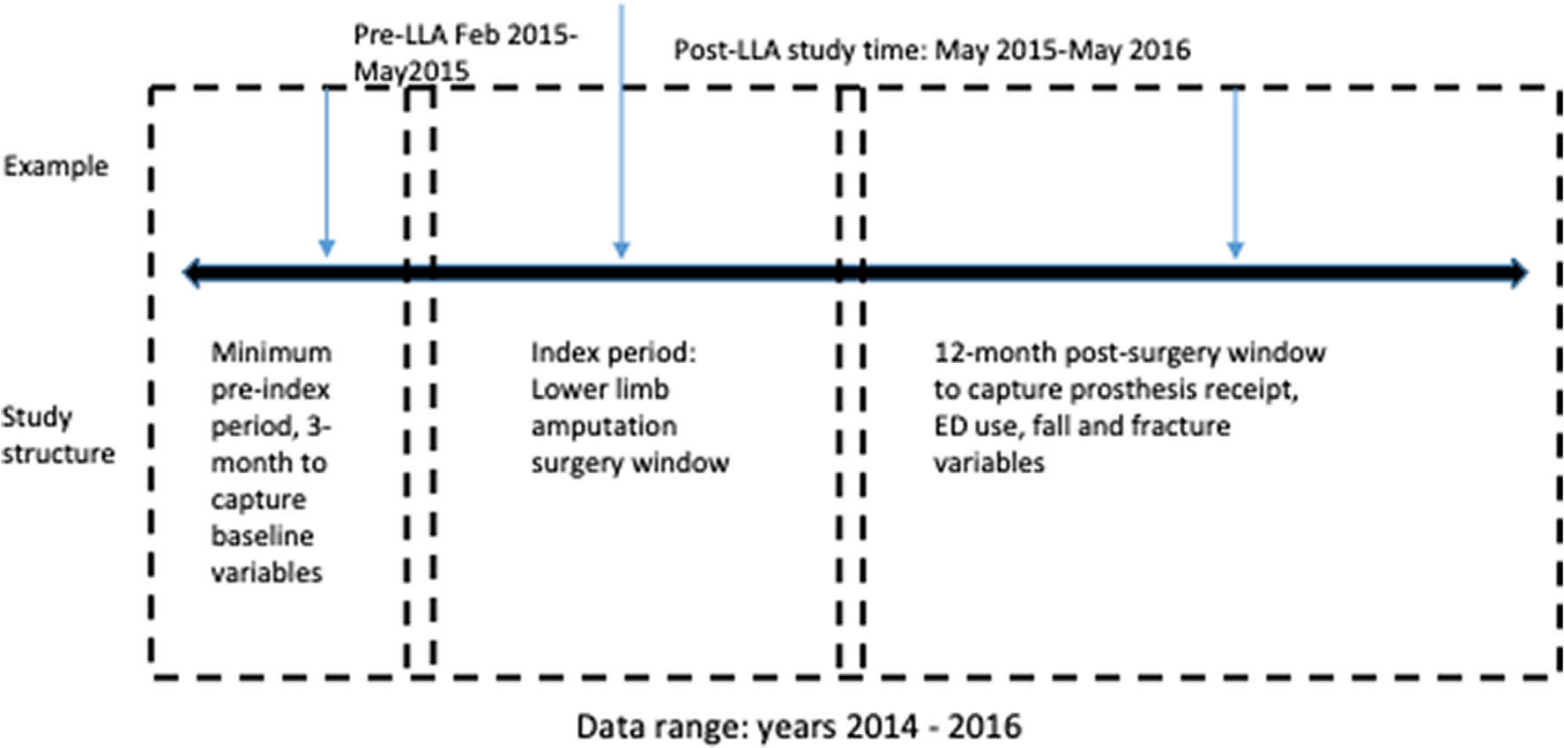
Structure of participant episode definitions and creation of sample. LLA = lower limb amputation; ED = emergency department.

Enrollees in one of the commercial plans contained within the Watson database who were 18 years or older with continuous health coverage for the 3‐year period (n = 1100) were eligible for the study. Next, inclusion was based on amputation procedure codes using the International Classification of Diseases (ICD) and Current Procedural Terminology (CPT) codes (ICD‐9 and ICD‐10 used due to time period crossing 2015) to identify all cases of LLA surgery. The index event was amputation surgery during 2014 through 2015 while allowing for 3 months of data pre‐index to capture baseline characteristics and 12 months of data postamputation surgery (Figure 1). Baseline characteristics include age, sex, amputation level, and diabetes/vascular status. Only patients with initial surgical LLA procedures that occurred within the index period were included in the final sample (n = 510; Figure 1). Time of prosthesis receipt was noted as the point where a prosthesis claim (ie, base prosthesis L‐code) appeared, which is the date of service the prosthesis was provided to the patient (Appendix A). Time was collapsed into mutually exclusive 3‐month categories or groups and treated as a categorical variable. Individuals who received a prosthesis within the 12 months postamputation were subsequently grouped based on time since amputation. Group time period breakdown was: 0 to 3 months postamputation, 4 to 6 months postamputation, 7 to 9 months postamputation, and 10 to 12 months postamputation. Individuals who did not receive a prosthesis within 12 months of their amputation were the final group: “no prosthesis.”

### Measures

The outcome variable, ED use, was defined as the presence of CPT codes billed for all‐cause ED services (99281 to 99285) on any claim that occurred after the amputation date up to 12 months postamputation. ED use was counted as a binary event, the first visit, or event occurrence postamputation was captured and counted based on procedure code. Fall/FRI was defined as the presence of diagnosis codes that met the criteria of fall (ICD‐9 and ICD‐10) in outpatient procedures that occurred after the amputation date up to 12 months postamputation (Appendix A). Fall/FRI was conceptualized as a proxy for functional limitation or reduced QoL for use in the second model as the fall required medical attention or health care utilization. Prosthesis receipt was determined by noting major lower limb prosthesis base codes billed after amputation surgery. Claims that included a prosthesis base code after amputation were flagged to have received a prosthesis and time elapsed postamputation noted. The prosthesis date of service is the date the prosthesis was delivered to the patient irrespective of the date billed. Presence of diabetes or vascular disease was identified based on ICD‐9 or ICD‐10 in claims any time after enrollees’ first admission, with the assumption that the disease persisted throughout the study period (Appendix A). Amputation level was determined based on diagnosis code. Amputation level was collapsed into two categories: above‐the‐knee (AKA) and below‐the‐knee (BKA). For example, amputations through the tibia (ie, transtibial) or distal to the knee were grouped as BKA. Amputation through‐the‐knee (knee disarticulation) and proximal, including at the level of the hip, were all grouped as AKA. Toe or partial‐foot amputations were excluded. There were no hemipelvectomy or further proximal levels of amputation.

### Analysis

Summary and descriptive statistics were calculated among the sample population based on receipt of prosthesis for population subgroups (Table 1). Characteristics are further described based on time from amputation surgery to delivery of prosthesis (Table 2). Chi‐square tests of independence or Wilcoxon‐Mann‐Whitney *U*‐tests were used to compare groups. Two‐tailed tests of significance with alpha levels set a priori at 0.05 were implemented in all statistical analyses. Next, bivariate logistic regression was used to model the crude association between outpatient fall/FRI and all‐cause ED use. Each independent variable was also analyzed in a bivariate model to measure the unadjusted association. Finally, multivariate logistic regression was used to calculate adjusted odds ratios (ORs) and 95% confidence intervals (CIs).

**Table 1:** Sample demographic characteristics and inferential statistics stratified by receipt of prosthesis

Demographic Characteristics	Receipt of Prosthesis		*P* value
	Yes	No	
Total population, n (%)	443 (87)	67 (13)	
Emergency department utilization			
Yes	131(83.4)	26 (16.6)	.13
No	312 (88.4)	41 (11.6)	
Fall after LLA			
Yes	396 (86.5)	62 (13.5)	.43
No	47 (90.4)	5 (9.6)	
Amputation level			
Transtibial or below knee	352 (91.4)	33 (8.6)	<.001*
Transfemoral or above knee	91 (72.8)	34 (27.2)	
Sex, n (%)			
Male	315 (88.7)	40 (11.3)	.06
Female	128 (82.6)	27 (17.4)	
Diabetes/vascular status			
Yes	290 (88.7)	37 (11.3)	.10
No	153 (83.6)	30 (16.4)	
Age, mean (SD)	52.5 (9.4)	52.1 (10.1)	.80

* Statistically significant at .05. LLA = lower limb amputation.

**Table 2:** Characteristics of patients based on time from amputation to receipt of prosthesis within 12 months, or no prosthesis

Demographic Characteristics	No Prosthesis		0‐3 mo		4‐6 mo		7‐9 mo		10‐12 mo		Total	
	N	%	N	%	N	%	N	%	N	%	N	%
Total population, n (%)	67	13.1	174	34.1	186	36.5	58	11.4	25	4.9	510	100
Emergency department utilization												
Yes	26	16.6	46	29.3	58	36.9	18	11.5	9	5.7	157	30.9
No	41	11.6	128	36.3	128	36.3	40	11.3	16	4.5	353	69.1
Amputation level												
Transtibial or below knee	33	8.5	141	36.6	150	39.0	46	12.0	15	3.9	385	75.4
Transfemoral or above knee	34	27.2	33	26.4	36	28.8	12	9.6	10	8.0	125	24.6
Sex, n (%)												
Male	40	11.2	142	40.0	121	34.0	41	11.5	14	3.9	355	70
Female	27	17.4	32	20.6	65	42.0	17	11.0	14	9.0	155	30
Diabetes/vascular status												
Yes	37	11.3	123	37.6	120	36.7	33	10.1	14	4.3	327	64.1
No	30	16.4	51	27.9	66	36.1	25	13.6	11	6.0	183	33.9
	**No Prosthesis**		**0‐3 mo**		**4‐6 mo**		**7‐9 mo**		**10‐12 mo**		**Total**	
Age, mean (SD)	52.1 ± 0.69		52.4 ± 0.69		52.4 ± 0.68		53.2 ± 1.2		50.7 ± 2.5		52.2 ± 0.42	

Group X = no prosthesis; Group A = 0‐3 months postamputation prosthesis receipt; Group B = 4‐6 months post; Group C = 7‐9 months post; Group D = 10‐12 months post.

In the first model to address hypothesis 1, the influence of timing of prosthesis receipt on the likelihood of all‐cause ED use was analyzed while controlling for age, sex, diabetes status, and amputation level. Time in 3‐month‐based categories between amputation surgery and receipt of prosthesis was included as a primary independent variable of interest (no prosthesis as referent category: 0 to 3 months, 4 to 6 months, 7 to 9 months, and 10 to 12 months). Unadjusted ORs and 95% CIs were obtained using bivariate logistic regression to provide a crude association for each variable with ED use. A multivariate logistic regression was model conducted and then a post hoc pairwise multiple comparisons was applied to evaluate the difference between time categories (Table 3). Subsequently, the predicted cumulative incidence function was applied to determine the predicted probability of ED use versus age while stratifying by time to prosthesis receipt groups.

**Table 3:** Logistic regression results for model 1

Variables	Model 1‐ Timing & ED Use	
	Unadjusted OR (95% CI)	Adjusted OR (95% CI)
Amputation level (AKA vs. BKA)	1.08 (0.70‐1.66)	1.09 (0.69‐1.73)
Sex (female vs. male)	0.93 (0.62‐1.40)	0.93 (0.61‐1.41)
Age	1.02 (0.99‐1.04)	1.01 (0.98‐1.03)
Diabetes/vascular status (no vs. yes)	0.68 (0.46‐1.02)	0.71 (0.47‐1.07)
Length of time, LLA to prosthesis		
0‐3 mo	0.47 (0.35‐1.42)	0.52 (0.28‐0.97)*
4‐6 mo	0.64 (0.26‐1.55)	0.68 (0.28‐1.26)
7‐9 mo	0.76 (0.34‐1.93)	0.69 (0.32‐1.48)
10‐12 mo	1.03 (0.30‐2.15)	0.92 (0.35‐2.41)
No prosthesis (Reference)	—	—

Unadjusted results represent the bivariate or crude relationship between the independent variable and outcome variable. The adjusted estimates are while controlling for covariates. ED = emergency department, AKA = above knee amputation, BKA = below knee amputation, LLA = lower limb amputation, OR = odds ratio, CI = confidence interval.

* Statistically significant based on 95% confidence interval.

A second model to address hypothesis 2, set the outcome of interest to ED use. Primary independent variables were fall/FRI and prosthesis receipt. Unadjusted OR and 95% CI were obtained using bivariate logistic regression to provide a crude association for each variable with ED use. Multivariate logistic regression was used to assess the fall/FRI association while adjusting for covariates, prosthesis receipt, and possible confounders, such as age, sex, and amputation level (Table 4). To assess confounding effects, each potential confounder was entered into a bivariate model separately. If the variable changed the magnitude of the OR compared to the crude OR by at least 10% it was considered a confounder.^14^

**Table 4:** Logistic regression results for model 2: the association of prosthesis receipt and fall/FRI with ED utilization

Variables	Model 2 ‐ FRI & ED Use	
	Unadjusted OR (95% CI)	Adjusted OR (95% CI)
Receipt of prosthesis (no vs. yes)	1.51 (0.89‐2.57)	1.52 (.87‐2.66)
Fall/FRI (yes vs. no)	3.13 (1.40‐7.12)	2.86 (1.23‐6.66)*
Amputation level (AKA vs. BKA)	1.08 (0.70‐1.66)	0.93 (0.58‐1.48)
Sex (female vs. male)	0.93 (.62‐1.40)	0.87 (0.56‐1.34)
Age	1.02 (0.99‐1.04)	1.01 (0.98‐1.03)
Diabetes/vascular status (no vs. yes)	0.68 (0.46‐1.02)	0.69 (0.45‐1.04)

Adjusted estimates are while controlling for covariates. FRI = fall related injury, AKA = above knee amputation, BKA = below knee amputation, LLA = lower limb amputation, OR = odds ratio, CI = confidence interval.

* Statistically significant based on 95% confidence interval.

Model assumptions and fitting were assessed using standard techniques, such as ROC curves. All analyses and data management were conducted using SAS 9.4 (Cary, NC).

## Results

### Descriptive Statistics

**Figure 2:**
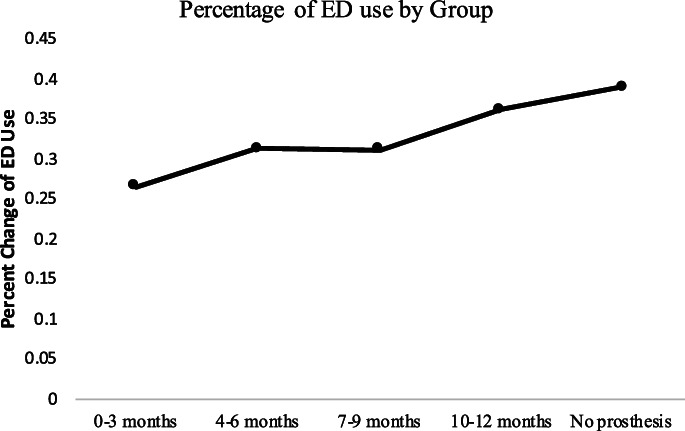
Comparison of percent of emergency department (ED) use that changes based on timing of prosthesis receipt proportional to group size. No prosthesis group, Group 0‐3 months, 4‐6 months, 7‐9 months, 10‐12 months postamputation prosthesis receipt.

Among the patients in the sample, 13% (67/510) did not receive a prosthesis within 12 months postamputation surgery (Table 1). Of those who received a prosthesis, 131 individuals (30%) utilized the ED postamputation as opposed to 26 individuals (39%) with no prosthesis (Table 1). Most of the individuals had a transtibial (below knee) amputation (75%). Three hundred twenty‐seven individuals (64%) had diabetes or vascular disease. The average age was 52 ‐years old (±9.4 years) and the majority of patients were male (70%). A simple comparison of the percent of ED use based on timing of prosthesis receipt groups demonstrates an upward pattern of increased ED use the longer it takes to get a prosthesis or not receiving one within 12 months (Figure 2).

### Model Results

After assessment for confounding effects, none of the potential confounding variables influenced the odds ratios (OR) by 10% or more. However, the decision was made to retain and control for the potential confounding variables in the final models based on strong previous literature identifying these variables as strong risk factors associated with falls/FRIs.^2^, ^15^

#### Model 1: ED Use and Timing of Prosthesis Receipt

The first model evaluated the impact of timing of prosthesis receipt against ED use while controlling for sex, age, diabetes/vascular status, and amputation level. Individuals who receive a prosthesis early, between 0 and 3 months, after LLA were 48% (OR 0.52, 95% CI 0.28 to 0.97) less likely to use the ED compared to the referent group, those who did not receive a prosthesis during the 12‐month post‐amputation period (Table 3). The other time periods were not significantly associated with predicting ED use as compared to the referent group. The covariates including sex, amputation level, age, and diabetes/vascular status were not significantly associated with ED use (Table 3).

**Figure 3:**
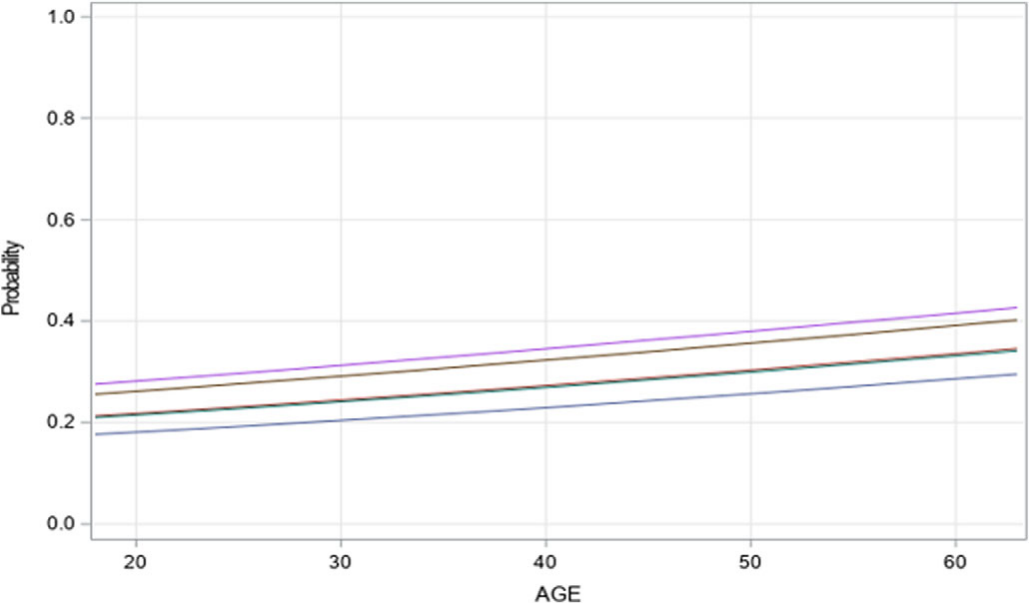
Predicted probability of ED use versus age stratified by time to prosthesis receipt. The purple line represents the “no prosthesis” referent group and is the highest line on the graph demonstrating a consistently higher probability of ED use. The gray line, the next one down, represents the 10‐12 month group for prosthesis receipt, whereas time periods 4‐6 months and 7‐9 months are the overlapping red and green lines. The lowest probability of ED use is seen with the blue line, which represents those who receive a prosthesis between 0 and 3 months.

The predicted probability graph (Figure 3), demonstrates the difference in risk of ED use versus age stratified by time of prosthesis receipt groups. At age 60, there is approximately a 40% increased probability of an individual with no prosthesis (purple line in Figure 3) to use the ED as compared to a 60‐year‐old who receives their prosthesis between 0 and 3 months. The increased risk of ED utilization appears to be a consistent trend the longer the time between LLA surgery and prosthesis receipt (Figure 3).

#### Model 2: Health Care Utilization/ED Use and Adverse Events

The second model assessed the factors associated with ED use while controlling for prosthesis receipt. Individuals who experienced an adverse event, defined as any fall/FRI after amputation surgery, had a 2.80 (95% CI 1.32 to 6.54) times increased odds of using the ED within the follow‐up period compared to those with no fall/FRI, while adjusting for covariates (Table 4). The covariates in model 2 were not significantly associated with ED use (Table 4).

## Discussion

Using claims data for commercially insured adults in the United States, these findings substantiate that (1) adults with no lower limb prosthesis within 12 months of amputation surgery are almost twice as likely to use the ED as compared to those who receive a prosthesis within 3 months, with the odds likely worsening with extended delays (Table 3), and (2) among those with LLA who experience a fall within 12 months of amputation surgery there is a 2.8 times increased odds of associated ED utilization (Table 4). This study included a representative sample population similar to estimates presented in previous work, with approximately 65% of lower limb amputee patients between 18 and 65 years of age male.^16^ Moreover, our sample is reflective of those with LLA and commercial insurance that highlights the potential value of a prosthesis in terms of health care utilization defined by all‐cause ED use at least once within 12 months postamputation. Those who receive a prosthesis early are less likely to use the ED and this indirectly may improve QoL.^7^ This study excludes determination of value in terms of return to work or as a measure of functional outcomes.

Our descriptive analysis suggests that with further time delay from surgery to receipt of prosthesis, the use of the ED increases as seen in the percent of ED use comparison (Figure 2). This is similar to our results presented in the multivariate analysis, with model 1 revealing a pattern of a graded decline in ORs with comparisons between individuals without a prosthesis and those individuals that were further removed from amputation (ie, 4 months up to 12 months post‐LLA) (Table 3). This observation is consistent with the expression that the earlier a prosthesis can be provided, the greater protection against the potentially preventable excess health care utilization or ED use as seen visually in the predicted probability graph (Figure 3) and therefore increased value associated with having the prosthesis.

### Early Prosthesis Intervention and Health Care Utilization

As the findings from model 1 suggest, earlier receipt of a prosthesis decreases health care utilization as measured by ED use. Earlier intervention with physical rehabilitation and lower limb prostheses has been suggested to improve patient functional mobility and therefore reduce health care utilization due to adverse events reinforcing prosthetic value.^17^ Pezzin et al (2004) conducted a survey, which included both patients with upper and lower limb amputations, and their findings suggested that a shorter time between amputation and receipt of a prosthesis improved patients’ reporting of satisfaction and increased use of devices. Furthermore, respondents from the survey reported an average time to receipt of prosthesis of 90 days. The reported shorter time from surgery to receipt of prosthesis and patients reporting improved function is consistent with the current study's findings that an earlier intervention may reduce health care utilization, such as ED visits, and reduce adverse events, thus increasing the economic value of a prosthesis. It has been suggested in other studies that there is a protective effect, such as reduced mortality, of initial prosthesis fitting.^18^ Mortality risk remains elevated among individuals with LLA beyond the initial 30 days, especially among adults who are not fit with a prosthesis within 6 to 12 months postsurgery.^18^ Individuals without a prosthesis will have more difficulty participating in physical therapy and mobility around the community, and have greater risk of fall or injury in the home. All of these factors can increase the incidence of adverse events, which lead to ED utilization and increased strain on the health care system.

The results of model 1 in this study did not indicate age, sex, and amputation level or diabetes/vascular disease status to be significant indicators for increased odds of ED use among people with LLA. The findings suggest that patient level factors such as age, sex, and cause of amputation should not create exemptions for certain patients to realize the benefits of early prosthesis fittings with regard to reduction in ED use. These findings are consistent with previous evidence that found that such factors were not necessarily restrictive to a patient's ability to achieve successful mobility with a prosthesis.^18^, ^19^

The provision and access to a prosthesis is a critical component of a person's rehabilitation after an LLA, as it is associated with a person's ability to return to ADLs, safety, and reintegrate into social or work routines.^5^, ^8^ It has become more important to provide evidence of benefit for provision of a prosthesis in order to ensure patients have appropriate access to a prosthesis.^8^, ^20^ This study further substantiates the value of a prosthesis while highlighting specifically that earlier receipt of a prosthesis impacts health care utilization by reducing the associated risk of ED use. This study's results demonstrate that having a prosthesis earlier is protective against using the ED while controlling for age, sex, amputation level, and diabetes status.

### Health Care Utilization Associated with Adverse Events

The current findings from model 2 are consistent with recent literature suggesting that individuals having a LLA incur increased use of healthcare services due to adverse events such as falls and fractures.^8^, ^21^ Dobson et al (2016) conducted a retrospective cohort study using Medicare beneficiaries, who were significantly older than our study group (sample mean age 73 years). They found that the number of falls or fractures were comparable or higher among those with LLA compared to the control group. It is worth noting that the Dobson et al study only included those subjects with LLA who had received a prosthesis within 12 months of amputation surgery. In contrast, the current study also included individuals without a prosthesis within 12 months of surgery for analysis.

Individuals who experienced a fall/FRI after discharge from amputation were almost 3 times more likely to use the ED in the current study. Falls are common among those with amputation, including injurious falls that result in need for medical treatment, which has been demonstrated in a previous study among community dwelling adults with LLA.^22^ Adults with LLAs are often described as an at‐risk population for increased health care utilization because of their increased association with chronic diseases and functional disability.^22^ The study by Mundell et al^23^ notes an association between transfemoral amputees and cardiovascular events. However, it was found that no change in risk occurred for those with or without a prosthesis.^23^ It is interesting to highlight that we found no effect of sex, as a few studies have found that women with limb loss have worse function or increased adverse events compared to men with limb loss.^22^, ^24^, ^25^ Yet, the current study differed from previous studies in that this study utilized claims of those with commercial insurance as opposed to relying on self‐report, which may have recall bias influencing the report of injurious falls. It is likely that other factors besides sex may be driving increased health care utilization within 12 months after LLA.

## Limitations

This study has several strengths, in particular the relatively large sample size for this specific population. In addition, findings are based on a nationally representative sample of commercial claims generalizable to the U.S. adult population with LLA who have commercial (private) health insurance. However, there are limitations of this study. Due to the nature of administrative data, it is not possible to differentiate the reasons some individuals took longer to receive a prosthesis or not at all. It is possible that a delayed fitting or functional limitations that contribute to falls may be associated with other health complications, lack of social support, certain payer policy restrictions, or another unseen complication that contributes to increased health care utilization. Future studies should attempt to determine such factors using clinical databases, or potentially registry data. However, understanding this should place greater emphasis on working to resolve issues seen to potentially delay provision of prosthetic rehabilitation so that patients are afforded the benefits of earlier receipt of their prosthesis.

The current study did not assess the events prior to ED use. Unfortunately the nature of claims data makes it difficult to determine events that may have precipitated or caused increased health care utilization. This study did not assess the potential difference in severity or frequency of ED use. Further exploration on the association of adverse events, falls/FRIs, and the subsequent acute care utilization with respect to frequency remains needed. Such work, as well as the current study, would be enhanced, if possible, to ascertain the functional status of patients. There are other confounding variables that influence both the timing of prosthesis receipt and falls/FRIs, such as poor general health and functional mobility. The receipt of a prosthesis, as captured by date of service, does not guarantee use of the prosthesis. However, a strength of using claims data includes accurate reflection of dates, such as the date a prosthesis was received, as opposed to relying on patient recall.

## Conclusion

The current study findings have valuable implications for clinical care and potentially policy. Earlier receipt of a prosthesis is associated with a reduced marginal odds of ED use. This is valuable in further consideration of the current study results showing a strong association between falls/FRIs and ED use, indicating that a prosthesis plays an important role in individual mobility and the potential to reduce preventable health care utilization. In light of previous work, which has noted the negative impact of ED utilization on QoL^7^ and overall quality of care, it is concluded that if an individual is provided a prosthesis earlier in the rehabilitation process after LLA then there is likely less economic burden and potential to improve patient outcomes. There is increased value and opportunity to avoid preventable health care utilization and further reductions in QoL.

**CME Question**

This retrospective study found that emergency department utilization after lower limb amputation was less likely:

- In below knee compared to above knee amputees
- Associated with falls within 12 months post amputation
- When prosthesis is received within 3 months of surgery
- In male versus female amputees of comparable age

**Answer online at** https://onlinelearning.aapmr.org/

## Supporting information

**Appendix A**: Amputation level determined by ICD‐9 or ICD‐10 procedure codes
