# Supplementary material for: The Role of Earlier Receipt of a Lower Limb Prosthesis on Emergency Department Utilization
Source: PM R. 2020 Dec 11;13(8):819–26. doi: 10.1002/pmrj.12504 (PMC8451817; doi:10.1002/pmrj.12504)
Supplement: Supplementary file 1 — Appendix A: Amputation level determined by ICD‐9 or ICD‐10 procedure codes [file PMRJ-13-819-s001.docx]

Appendix A

Amputation level determined by ICD-9 or ICD-10 procedure codes

| Amputation level | ICD-9 | ICD-10 | CPT code |
| --- | --- | --- | --- |
| Transtibial or below knee | 84.1 | 0Y6H0Z1 | 27880 |
|  | 84.12 | 0Y6H0Z2 | 27881 |
|  | 84.13 | 0Y6H0Z3 | 27884 |
|  | 84.14 | 0Y6J0Z1 | 27886 |
|  | 84.15 | 0Y6J0Z2 | 27888 |
|  |  | 0Y6J0Z3 | 27889 |
|  |  | 0Y6M0Z0 |  |
|  |  | 0Y6N0Z0 |  |
| Transfemoral or above knee | 84.16 | 0Y670ZZ | 27590 |
|  | 84.17 | 0Y680ZZ | 27591 |
|  | 84.18 | 0Y6C0Z1 | 27592 |
|  | 84.19 | 0Y6C0Z2 | 27594 |
|  |  | 0Y6C0Z3 | 27596 |
|  |  | 0Y6D0Z1 | 27598 |
|  |  | 0Y6D0Z2 |  |
|  |  | 0Y6D0Z3 |  |
|  |  | 0Y6F0ZZ |  |
|  |  | 0Y6G0ZZ |  |

Diabetes or vascular disease status identified as claims that included a diagnosis of diabetes mellitus type II, (specified or unspecified and with or without complications), atherosclerosis of the extremities, or peripheral vascular disease

| Condition | ICD-9 | ICD-10 |
| --- | --- | --- |
| Diabetes | 250.00-250.99 | E11.8-11.9 |
|  |  | E11.620-11.628 |
| Peripheral vascular disease/Atherosclerosis | 440.0 | I70.0-79.9 |
|  | 443.9 |  |
|  |  |  |

Base prosthesis L-codes to determine receipt of prosthesis

| Amputation level | HCPC/L-Code |
| --- | --- |
| Transtibial or below knee | L5050, L5060, L5100, L5105, L5301 |
|  | L5050, L5060, L5100, L5105, L5301 |
|  |  |
| Transfemoral or above knee | L5150, L5160, L5200, L5210, L5220, L5230, L5250, L5270, L5280, L5312, L5321, L5331, L5341 |
|  |  |
|  |  |

Fall diagnosis codes in outpatient services

| Condition | ICD-9 | ICD-10 |
| --- | --- | --- |
| Fall | E880.00-E888.9 | W00-W19 |
|  |  |  |
